# AI-based selection of tumor regions for genomic profiling in neuropathology

**DOI:** 10.1093/noajnl/vdag157

**Published:** 2026-06-12

**Authors:** Narmin Ghaffari Laleh, Lukas Friedrich, Fuat Kaan Aras, Katherine J Hewitt, Leonille Schweizer, Zunamys I Carrero, Dilan Savran, Daniel Haag, Silvia Barbosa, Felix Sahm, Jakob Nikolas Kather

**Affiliations:** Else Kroener Fresenius Center for Digital Health, Faculty of Medicine and University Hospital Carl Gustav Carus, TUD Dresden University of Technology, Dresden, Germany; Frankfurt Cancer Institute (FCI), Frankfurt am Main, Germany; Frankfurt Cancer Institute (FCI), Frankfurt am Main, Germany; Else Kroener Fresenius Center for Digital Health, Faculty of Medicine and University Hospital Carl Gustav Carus, TUD Dresden University of Technology, Dresden, Germany; German Cancer Consortium (DKTK), Partner Site Frankfurt/Mainz, German Cancer Research Center (DKFZ), Heidelberg, Germany; Institute of Neurology (Edinger Institute), University Hospital Frankfurt, Goethe University, Frankfurt am Main, Germany; Medical Oncology, National Center for Tumor Diseases (NCT), University Hospital Heidelberg, Heidelberg, Germany; Else Kroener Fresenius Center for Digital Health, Faculty of Medicine and University Hospital Carl Gustav Carus, TUD Dresden University of Technology, Dresden, Germany; Frankfurt Cancer Institute (FCI), Frankfurt am Main, Germany; Frankfurt Cancer Institute (FCI), Frankfurt am Main, Germany; Dept. of Neuropathology, University Hospital Heidelberg, Heidelberg Germany and CCU Neuropathology, German Consortium for Translational Cancer Research (DKTK), German Cancer Research Center (DKFZ), Heidelberg, Germany; Frankfurt Cancer Institute (FCI), Frankfurt am Main, Germany; Else Kroener Fresenius Center for Digital Health, Faculty of Medicine and University Hospital Carl Gustav Carus, TUD Dresden University of Technology, Dresden, Germany; Dept. of Neuropathology, University Hospital Heidelberg, Heidelberg Germany and CCU Neuropathology, German Consortium for Translational Cancer Research (DKTK), German Cancer Research Center (DKFZ), Heidelberg, Germany; Department of Medicine I, Faculty of Medicine and University Hospital Carl Gustav Carus, TUD Dresden University of Technology, Dresden, Germany; Pathology & Data Analytics, Leeds Institute of Medical Research at St James’s, University of Leeds, Leeds, United Kingdom

**Keywords:** glioma, molecular diagnostics, multiple instance learning, tumor region segmentation, weakly supervised learning

## Abstract

Automating pathology workflows with deep learning is increasingly feasible and clinically relevant. We present an AI-based method that identifies diagnostically relevant areas directly from H&E-stained slides, trained on 250 glioma cases using sparse, incomplete annotations. First, we show that attention-based multiple instance learning achieves accurate predictions despite noisy labels, easing the annotation burden. Second, the model highlights tumor regions with high cellularity or grade, offering reproducible guidance for tissue selection. In a prospective evaluation, AI-selected regions achieved a mean Dice score of 0.743 [±0.077], supporting integration into neuropathology workflows as reliable guidance for molecular diagnostics.

Recent advances in deep learning and the availability of large-scale histopathology datasets have made it increasingly feasible to automate pathology workflows.^1^ This is particularly relevant in neuropathology, where brain tumors are heterogeneous, infiltrative, and often contain variable tumor cell content ranging from 5% to 95%.^2^ Deep learning models have shown promising performance on several tasks using H&E-stained whole slide images (WSIs), including classification of methylation-based CNS tumor subtypes,^3^ WHO subtype prediction,^4^ and detection of diagnostic alterations.^5^

However, a major challenge in applying such methods in clinical workflows lies in identifying the diagnostically most informative region of interest (ROI). For downstream molecular analyses, such as methylation arrays or next-generation sequencing, only one tissue region is sampled, typically by 1-3 mm punches or by scratching FFPE sections with or without microdissection, depending on histologically estimated tumor cell content.^2^ Selecting a representative tumor region is critical to avoid under-sampling, which may result in missed diagnoses, failure to detect actionable mutations, or uninterpretable molecular profiles that require testing.

Currently, ROI selection is performed manually by a neuropathologist, a process that is subjective, labor-intensive, and increasingly unsustainable given shortages of trained specialists.^5^ Existing computational approaches for automated ROI detection in WSIs, including rule-based cellularity tools such as QuPath^6^ and weakly supervised methods,^7^ have shown promise but were not specifically developed or validated for the complex tissue heterogeneity characteristic of brain tumors. Automating this step could improve consistency, reduce labor, and enable cross-center harmonization of results. However, training AI models for this task typically requires dense annotations or pixel-level labels across large cohorts, which are too time-intensive to be practical.

In this study, we present a deep learning approach that achieves 2 main goals: (1) it is trained using sparse, intentionally imperfect annotations (“noisy labels”) from trainee neuropathologists and (2) it produces ROI heatmaps that can guide tissue selection for molecular profiling. We hypothesized that even coarse labels would be sufficient to train a model capable of distinguishing diagnostically useful tumor regions from non-informative regions, motivated by the success of weakly supervised learning in histopathology.^8^

Using 250 H&E-stained WSIs from 250 patients (Table S1), we trained a weakly supervised multiple instance learning (MIL) model (Figure 1A). For each case, 2 trainee neuropathologists (FK, LF) marked tumor regions quickly and without attempting exhaustive delineation. For each patient, we extracted tiles within these annotations (Patient_N_ROI) and tiles from the remaining tissue (Patient_N_nonROI, Figure 1B). These were used to create positive and negative bags of tile-level embeddings, generated using a pretrained histology encoder (virchow_2),^9^ which has been shown to outperform alternative foundation models across a wide range of downstream tasks.^10^ This setup resulted in 500 embedding bags across all cases (Supplementary Methods).

**Figure 1.**
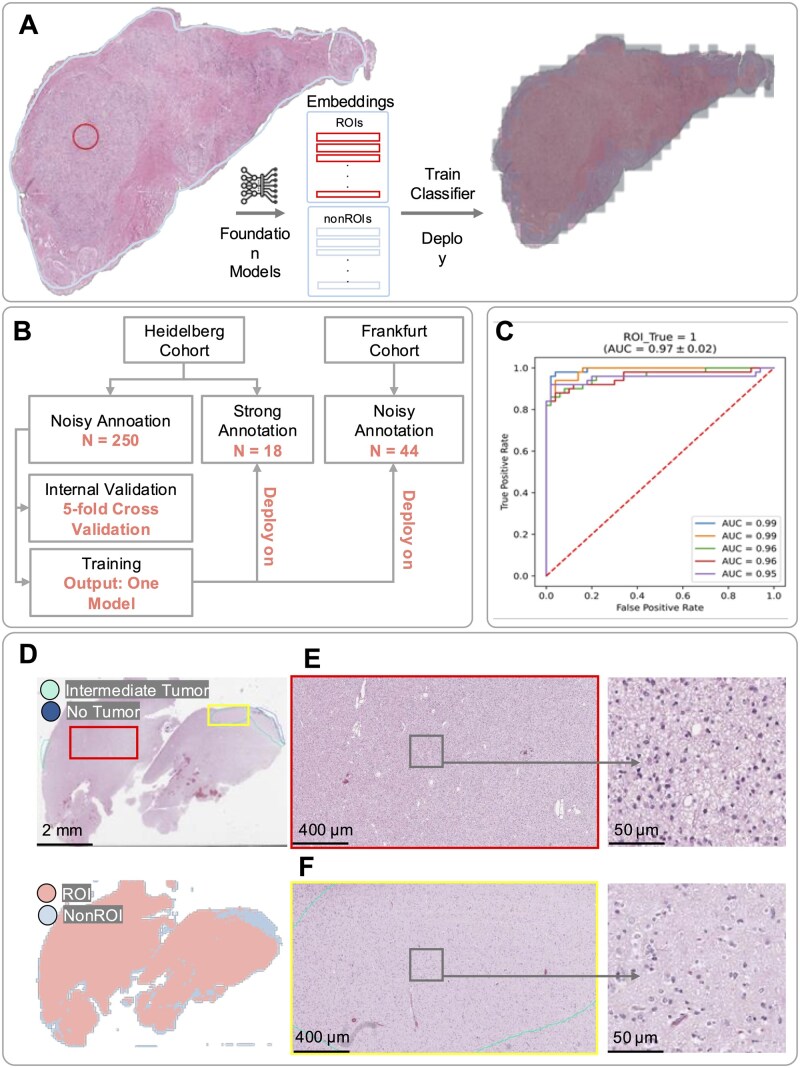
Workflow for automated ROI detection. (A) The pipeline begins with a whole slide image (WSI), from which candidate regions of interest (ROIs) are extracted. ROI and non-ROI tiles are embedded and passed through a neural network model for classification. (B) The model is trained using 250 WSIs and then deployed on 2 external datasets (*n* = 18 and *n* = 44). (C) The AUROC of 5-fold cross-validation demonstrated high performance of the ROI detection model with an average AUC of 0.97 ± 0.02. (D) Representative whole slide image with expert annotations indicating intermediate tumor and non-tumor regions, alongside the corresponding AI-generated heatmap showing predicted ROI (red) and non-ROI (blue) regions. (E) High-magnification view of the tumor region (red box in D), showing increased cellularity characteristic of solid tumor tissue (400 µm overview with 50 µm inset). (F) High-magnification view of the intermediate tumor region (yellow box in D), showing infiltrative tumor morphology with lower cellularity (400 µm overview with 50 µm inset).

The MIL model was trained to classify embedding bags using 5-fold cross-validation, ensuring that ROI and non-ROI bags from each case remained in the same data split (https://github.com/KatherLab/marugoto/tree/main). The model achieved an average AUROC of 0.97 [±0.023 (95% CI)] across folds (Figure 1C), confirming strong discriminatory performance. Following cross-validation, we trained a final model on the full training cohort and evaluated it on 2 external validation sets (*n* = 18 and *n* = 44). The model was used to generate ROI heatmaps by classifying tile embeddings across the WSI (Figure 1D). This yielded segmentation maps that highlight regions most suitable for tissue punching and downstream molecular analysis (Figure S1).

To quantitatively assess the model’s localization performance, detailed ROI maps were generated for a subset of the validation cohort (*n* = 18). Binary masks were created for tumor, intermediate tumor, and non-tumor regions (Figure 1D). Dice similarity coefficients were calculated for predicted ROI regions versus these masks, yielding a mean Dice score of 0.743 [±0.162] for tumor-only regions and 0.764 [±0.154] when including both tumor and intermediate tumor areas. Additionally, a neuropathologist (LF) reviewed the heatmaps for all external cases and scored them on a 0 to 4 scale based on the proportion of tumor highlighted (0: ROI highlighted non-tumor tissue only; 1: ROI predominantly highlighted non-tumor tissue; 2: ROI highlighted approximately 50% tumor and 50% non-tumor tissue; 3: ROI predominantly highlighted tumor tissue; 4: ROI highlighted tumor tissue only) (Figure S2). The model achieved a score of 3 or 4 in 95% of cases, indicating strong alignment with expert judgment (score 0—0%, score 1—0%, score 2—5%, score 3—35%, and score 4—60%). While isolated cases showed reduced discrimination between solid tumor and infiltrative regions, the distinction between infiltration and non-tumor tissue was consistently excellent (Table S2).

Finally, to assess clinical utility, we performed differential methylation analysis in 4 additional cases, comparing 1 ROI and 1 non-ROI punch selected by the model per case (Figure S3A). In 1 case the non-ROI sample had a non-evaluable methylation score. Two cases showed CNV plots with lower quality, whereas 1 case yielded equal results. ROI punches consistently produced correct methylation classifications and high-quality molecular profiles (DNA methylation classification results). Detailed DNA methylation classification results and CNV profiles for all 4 cases are available via the link provided in the Data Availability section.

Together, these findings demonstrate that a simple deep learning model trained in a weakly supervised manner can effectively localize diagnostically relevant tumor regions in brain tumors. This study focuses on feasibility rather than exhaustive benchmarking against alternative methods. Our results show that coarse, “noisy” annotations are sufficient to train useful models, substantially reducing annotation effort while maintaining robust performance. Given that high-quality, pixel-level annotations are rarely available at scale in routine practice, this supports a more realistic and scalable approach. While more precise annotations may further improve quantitative metrics, our findings indicate that fast, coarse annotations can still achieve clinically meaningful ROI selection. Automated ROI selection could serve as a foundation for integrating AI into the broader diagnostic workflow in neuropathology, including tumor classification and molecular testing, tumor grading, spatial transcriptomics, and efficient annotation of large histopathology datasets. In edge cases with low or absent tumor content (Figure S3B), the model is expected to produce predominantly non-ROI heatmaps; however, pathologist review remains essential in such scenarios to confirm whether the available slide is suitable for molecular analysis or whether an alternative section should be selected. This consideration could be further supported in future work by incorporating a confidence-based ROI area metric to objectively flag cases with insufficient tumor tissue. As the model is trained on real neuropathology cases rather than engineered features, it likely captures a broader spectrum of morphological characteristics beyond cellularity, offering potential advantages over rule-based computational approaches in complex infiltrative settings. Furthermore, extending the pipeline to process multiple slides per case simultaneously would reduce the need for initial slide selection by the pathologist. Automated ranking of detected regions based on histological high-grade features, such as mitotic activity, could further streamline the diagnostic workflow and decrease pathologist burden in routine practice. The model is designed to distinguish diagnostically relevant from non-relevant regions and does not explicitly quantify tumor cellularity or rank multiple tumor regions with different histological characteristics.

## Supplementary Material

vdag157_Supplementary_Data

## Data Availability

The data supporting the findings of this study are available from the corresponding author upon reasonable request. The DNA methylation classification results are publicly available at: https://drive.google.com/file/d/1gUET3Nz3WY4Ou8M9SaMbo79rN9CmsKWT/view? usp=drive_link.
