# Supplementary material for: AI-based selection of tumor regions for genomic profiling in neuropathology
Source: Neurooncol Adv. 2026 Jun 12;8(1):vdag157. doi: 10.1093/noajnl/vdag157 (PMC13332501; doi:10.1093/noajnl/vdag157)
Supplement: vdag157_Supplementary_Data [file vdag157_supplementary_data.zip › Supplementary Methods - clean.docx]

## **Supplementary Methods**

### **Annotation Procedure**

For each whole slide image (WSI), two trainee neuropathologists (FK, LF) performed manual annotations to identify representative tumor regions. For cases with multiple available slides, a slide containing tumor tissue was prioritized for selection, reflecting standard clinical practice. Annotations were intentionally generated in a rapid and non-exhaustive manner, without detailed boundary delineation, to reflect a realistic clinical workflow. Only a binary distinction (tumor vs. non-tumor) was used for model training. This design choice was motivated by the observation that high-quality, pixel-level annotations are rarely available at scale in routine neuropathology, whereas coarse annotations can be generated efficiently and are more widely accessible, addressing a key practical limitation for large-scale model development.

### **Tile extraction and preprocessing**

Whole-slide images (WSIs) were tessellated into non-overlapping image patches of 224 × 224 pixels (corresponding to approximately 256 µm at the given resolution), consistent with common preprocessing pipelines in computational pathology. Tiles containing predominantly background were excluded using Canny edge detection. No stain normalization was applied during preprocessing.

### **Feature extraction**

Feature embeddings were extracted from image tiles using Virchow2, a pretrained vision transformer–based foundation model trained on large-scale histopathology data. The model encodes tiles into high-dimensional representations capturing relevant morphological features and has been shown to generalize across diverse tissue types. In this study, Virchow2 was used as a fixed feature extractor without additional fine-tuning.

### **Multiple instance learning (MIL) framework**

A weakly supervised multiple instance learning (MIL) approach was used for model training. For each case, two bags of embeddings were constructed: one containing tiles from annotated ROI regions and one containing tiles from non-ROI regions. Each bag was assigned a binary label (ROI vs. non-ROI). This resulted in a total of 500 bags across 250 patients. The MIL model was trained to distinguish between these classes based on aggregated tile-level information, without requiring pixel-level supervision.

### **Training and validation**

Model training was performed using five-fold cross-validation with patient-level splitting on 250 cases, resulting in a total of 500 bags (one ROI and one non-ROI bag per case). ROI and non-ROI bags from the same patient were always assigned to the same fold to prevent data leakage. After cross-validation, a final model was trained on the full dataset (n = 250) and evaluated on two independent external validation cohorts (n = 18 and n = 44).

### **Evaluation and ROI heatmap generation**

For inference, tile-level embeddings were classified across entire WSIs to generate spatial probability maps, which were visualized as ROI heatmaps. To assess localization performance, a subset of validation cases (n = 18) was annotated in more detail, including three categories: tumor, intermediate tumor, and non-tumor. These refined annotations were used exclusively for evaluation and were not part of the training process. Overlap between predicted ROI regions and ground truth annotations was quantified using Dice similarity coefficients. In addition, qualitative assessment was performed by an expert neuropathologist to evaluate clinical relevance of the predicted regions.
