# Supplementary material for: AI-based selection of tumor regions for genomic profiling in neuropathology
Source: Neurooncol Adv. 2026 Jun 12;8(1):vdag157. doi: 10.1093/noajnl/vdag157 (PMC13332501; doi:10.1093/noajnl/vdag157)
Supplement: vdag157_Supplementary_Data [file vdag157_supplementary_data.zip › Supplementary Table 2.docx]

| **Rating** | **Heidelberg Cohort** | **Frankfurt Cohort** | **Merged** |
| --- | --- | --- | --- |
| 0 | 0 (0%) | 0 (0%) | 0 (0%) |
| 1 | 0 (0%) | 0 (0%) | 0 (0%) |
| 2 | 1 (6%) | 2 (5%) | 3 (5%) |
| 3 | 8 (44%) | 14 (32%) | 22 (35%) |
| 4 | 9 (50%) | 28 (64%) | 37 (60%) |
| Total | 18 | 44 | 62 |
