# Supplementary material for: AI-based selection of tumor regions for genomic profiling in neuropathology
Source: Neurooncol Adv. 2026 Jun 12;8(1):vdag157. doi: 10.1093/noajnl/vdag157 (PMC13332501; doi:10.1093/noajnl/vdag157)
Supplement: vdag157_Supplementary_Data [file vdag157_supplementary_data.zip › Supplementary Table and Figure Legends - clean.docx]

# **Supplementary Figure Legends**

**"Supplementary Figure 1** - Representative examples of expert annotations and model-predicted heatmaps. **A)** Whole slide image with expert annotations for a case with fragmented tissue sections, showing tumor , intermediate tumor, and non-tumor regions, with corresponding high-magnification insets (50 µm) illustrating the histological appearance of each annotated category. **B)** Whole slide image of a more complex case with multiple tissue fragments, showing expert annotations across all three categories alongside representative high-magnification insets (50 µm) for each region type. **C)** ROI heatmap generated by the trained MIL model for the case shown in A , with red indicating predicted ROI and blue indicating predicted non-ROI regions. D) ROI heatmap generated by the trained MIL model for the case shown in B, demonstrating the model's ability to identify diagnostically relevant tumor regions across heterogeneous tissue distributions.

**Supplementary Figure 2** - Representative examples of neuropathologist-scored ROI heatmaps from the Frankfurt cohort. For each case, the H&E-stained whole slide image (left) is shown alongside the corresponding model-predicted heatmap (right), with red indicating predicted ROI and blue indicating predicted non-ROI regions. Cases are shown for three scoring levels: A) Score 4: the model successfully identified tumor regions across the slide; note that some out-of-focus areas were not evaluable by the algorithm. B) Score 3: the model predominantly highlighted tumor tissue, though some infiltration zones were misclassified as tumor. C) Score 2: the model highlighted approximately equal proportions of tumor and non-tumor tissue; this case contained a large area of necrotic tissue, which contributed to reduced discrimination performance. Scores were assigned by an expert neuropathologist on a scale of 0-4, where 0 indicates ROI highlighting non-tumor tissue only and 4 indicates ROI highlighting tumor tissue only.

**Supplementary Figure 3** - Histological and model prediction examples for cases selected for methylation analysis. A) High-magnification views (60 µm) of model-selected tumor (left) and non-tumor (right) regions for all four cases submitted for differential methylation analysis. Tumor regions show characteristic increased cellularity and nuclear atypia, while non-tumor regions display predominantly non-neoplastic tissue including vascular structures, necrosis, and connective tissue. B) Corresponding model-predicted heatmap, with red indicating predicted ROI and blue indicating predicted non-ROI regions. The model successfully restricted its predictions to the limited tumor-containing areas, producing a predominantly blue heatmap reflective of the low tumor content. This example illustrates that in low-tumor settings the model does not randomly flag the entire slide as ROI.

#

# **Supplementary Table Legends**

**Supplementary Table 1** - Clinicopathological characteristics of the training and test cohorts. N, number of cases. WHO Grade was assigned according to the WHO Classification of Central Nervous System Tumors. Tumor cellularity variation was assessed histologically and recorded as homogeneous or heterogeneous. Categorical location refers to the anatomical region of the tumor. Missing values indicate cases where data were unavailable.

**Supplementary Table 2** - Overview of the scoring statistics for both test cohorts. 0: ROI highlighted non-tumor tissue only; 1: ROI predominantly highlighted non-tumor tissue; 2: ROI highlighted approximately 50% tumor and 50% non-tumor tissue; 3: ROI predominantly highlighted tumor tissue; 4: ROI highlighted tumor tissue only.
