# Supplementary material for: AI-based selection of tumor regions for genomic profiling in neuropathology
Source: Neurooncol Adv. 2026 Jun 12;8(1):vdag157. doi: 10.1093/noajnl/vdag157 (PMC13332501; doi:10.1093/noajnl/vdag157)
Supplement: vdag157_Supplementary_Data [file vdag157_supplementary_data.zip › Supplementary Table 1.docx]

| **Variable** | **Subcategory** | **Heidelberg (Train)** | **Heidelberg (Test)** | **Frankfurt (Test)** |
| --- | --- | --- | --- | --- |
| N | - | 250 | 18 | 44 |
| Diagnosis | Meningioma | 59 (23.6%) | 2 (11.1%) | 4 (9.1%) |
|  | Glioblastoma IDH-wildtype | 43 (17.2%) | 4 (22.2%) | 15 (34.1%) |
|  | Astrocytoma, IDH-mutant | 16 (6.4%) | 2 (11.1%) | 5 (11.4%) |
|  | Neurinoma | 16 (6.4%) | 1 (5.6%) | 2 (4.5%) |
|  | Carcinoma metastasis | 14 (5.6%) | 2 (11.1%) | 0 (0%) |
|  | Pituitary adenoma | 14 (5.6%) | 0 (0%) | 1 (2.3%) |
|  | Oligodendroglioma, IDH-mutant, 1p/19q codeleted | 11 (4.4%) | 1 (5.6%) | 2 (4.5%) |
|  | Pilocytic astrocytoma | 9 (3.6%) | 2 (11.1%) | 2 (4.5%) |
|  | Other Diagnosis | 68 (27.2%) | 4 (22.2%) | 13 (29.5%) |
| WHO Grade | 1 | 85 (34.0%) | 5 (27.8%) | 9 (20.5%) |
|  | 2 | 40 (16.0%) | 4 (22.2%) | 11 (25.0%) |
|  | 3 | 13 (5.2%) | 3 (16.7%) | 1 (2.3%) |
|  | 4 | 61 (24.4%) | 4 (22.2%) | 18 (40.9%) |
|  | Missing | 51 (20.4%) | 2 (11.1%) | 5 (11.4%) |
| Tumor cellularity variation | Heterogenous | 95 (38.0%) | 8 (44.4%) | - |
|  | Homogenous | 152 (60.8%) | 10 (55.6%) | - |
|  | Missing | 3 (1.2%) | 0 (0%) | - |
| Categorized Location | Supratentorial | 157 (62.8%) | 13 (72.2%) | 33 (75.0%) |
|  | Infratentorial | 36 (14.4%) | 2 (11.1%) | 3 (6.8%) |
|  | Spinal | 35 (14.0%) | 1 (5.6%) | 5 (11.4%) |
|  | Midline | 13 (5.2%) | 0 (0%) | 1 (2.3%) |
|  | Peripheral | 5 (2.0%) | 0 (0%) | 0 (0%) |
|  | Missing | 3 (1.2%) | 2 (11.1%) | 2 (4.5%) |
