# Supplementary figures and images for: AI-based selection of tumor regions for genomic profiling in neuropathology
Source: Neurooncol Adv. 2026 Jun 12;8(1):vdag157. doi: 10.1093/noajnl/vdag157 (PMC13332501; doi:10.1093/noajnl/vdag157)

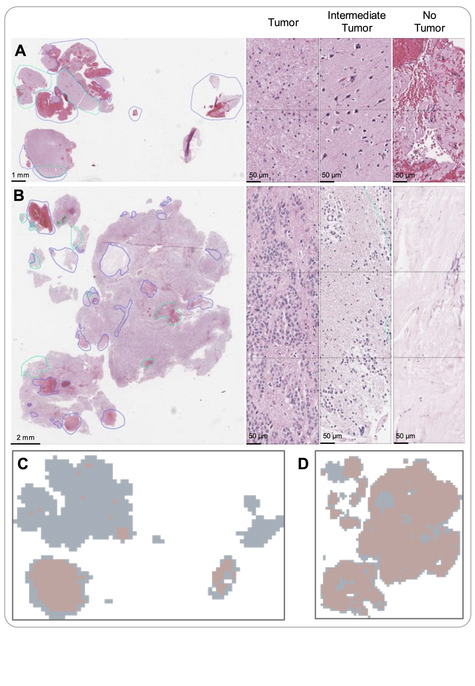

Supplement: vdag157_Supplementary_Data [file vdag157_supplementary_data.zip › Suppl_Figure_1.tiff]

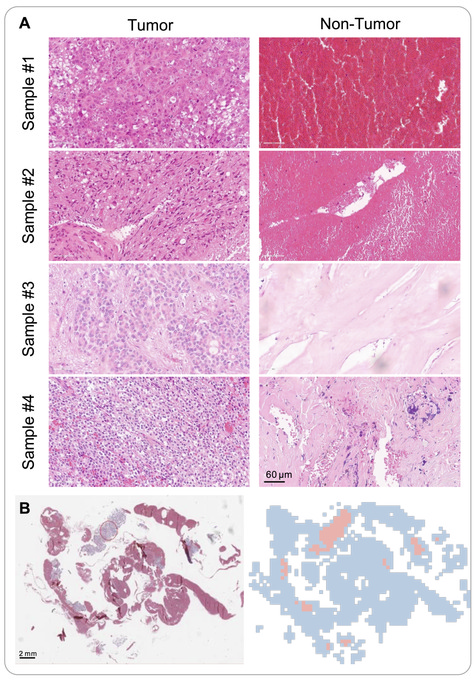

Supplement: vdag157_Supplementary_Data [file vdag157_supplementary_data.zip › Suppl_Figure_3.tiff]

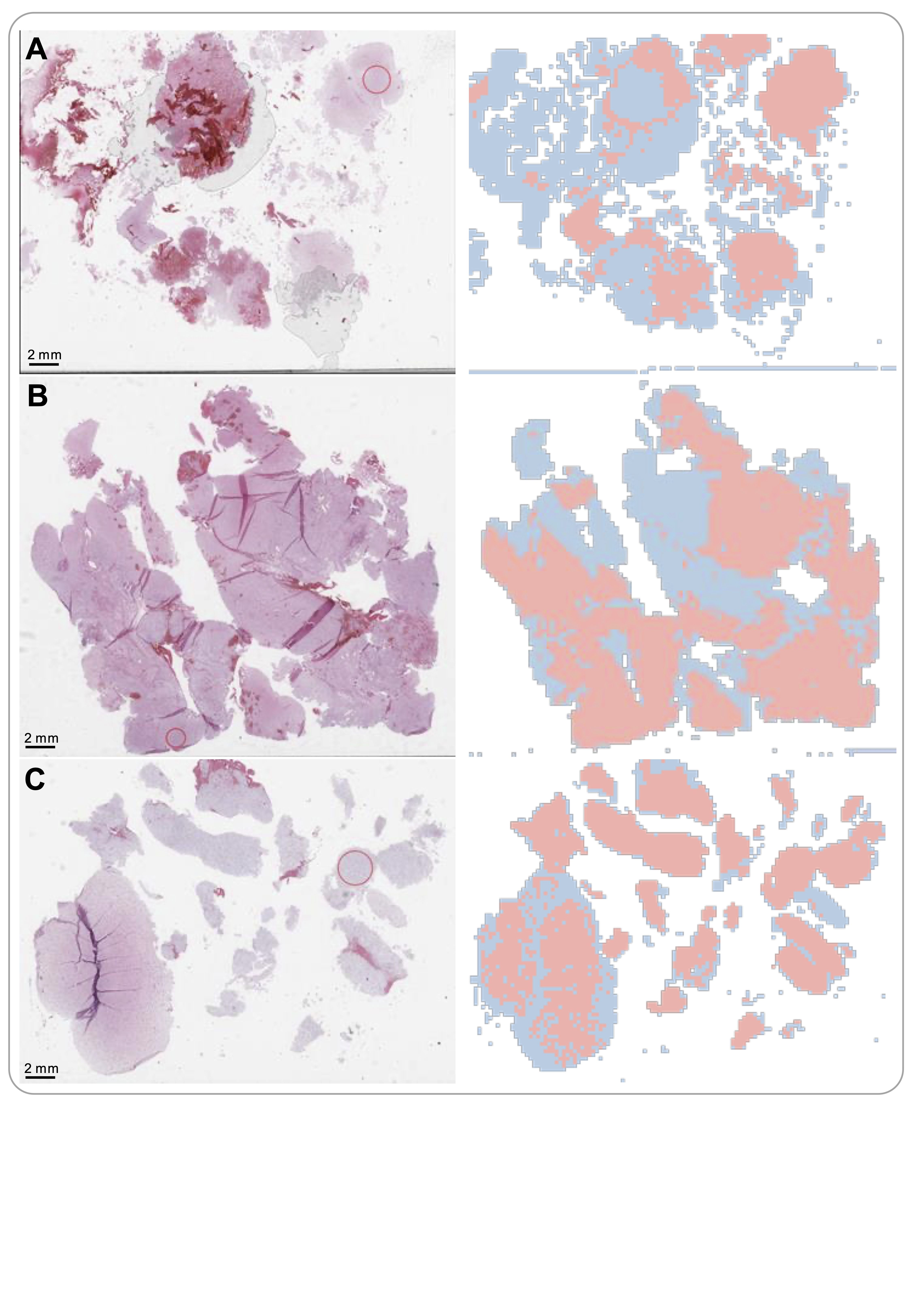

Supplement: vdag157_Supplementary_Data [file vdag157_supplementary_data.zip › Suppl_Figure_2.tiff]
